# Nonconformity of Medicine

**Published:** 1910-07-09

**Authors:** 


### Editor's Letter-Box.

### NONCONFORMITY OF MEDICINE.

*To the Editor of* The Hospital.

Sir,—Are you not rather hard upon nonconformity in medicine?

Emerson says, "Who would be a man must be a nonconformist," by which I guess he means a manly man, one who has not only the genius to arrive at advanced opinions but the courage to support them.

Take your horrid example, Mr. G. B. Shaw. One would be disposed to imagine there must be something very wrong with that gentleman, whereas really there is more good in him than the men in the street have yet had the wit to discover. Fortunately he has the additional saving grace of humour, which enables him to make life a comedy which most nonconformists find a tragedy. You say Mr. G. B. Shaw is a Socialist. Is it senseless or wicked for any man to wish for a well-paid public appointment? All such are Socialists in spite of themselves. He is also an Agnostic! Is it well to swallow all the dogmas of the Churches? Everyone who does not is dubbed an Agnostic. Then he is a feminist! Is it wicked to imagine women may have legal rights at the hustings as well as men? All such are feminists! Moreover, he is a teetotaler! Is it unhygienic to be a total abstainer when life insurance offices quote special lower rates for such? All who say so are ignorant and unscientific. And so on. May one privately admit all these things, but find it necessary publicly to denounce them? You are evidently a devotee of the commonplace—of the usual—and full of suspicion of the pioneers of progress. You consider that medical nonconformists "have hindered the cause of humanity sadly by their preference for anybody who opposes recognised medical teaching." The question arises, "How long is 'recognised' medical teaching worthy of recognition?" Forty years ago gross drugging, phlebotomy and blistering and leeching were "recognised," and woe did betide the nonconformists who dared deride their virtues. Was it such a dire offence or danger to the cause of humanity to dare to break with such traditions? Were not Pasteur and Lister, our most daring innovators, most disgraceful nonconformists? We need not less but more nonconformity. The dogmas of the professors of our school and college days should not too long wrap us round. We want more private research and scientific adventure—not less; more genius, more originality, and less worship of commonplace recognised authority. Who of us can say there are no more worlds left to discover, that wisdom must die with the recognised authorities? J. M. S.

